# Improving the effectiveness of Field Epidemiology Training Programs: characteristics that facilitated effective response to the COVID-19 pandemic in Uganda

**DOI:** 10.1186/s12913-022-08781-x

**Published:** 2022-12-16

**Authors:** Julie R. Harris, Daniel Kadobera, Benon Kwesiga, Steven N. Kabwama, Lilian Bulage, Henry B. Kyobe, Atek A. Kagirita, Henry G. Mwebesa, Rhoda K. Wanyenze, Lisa J. Nelson, Amy L. Boore, Alex Riolexus Ario

**Affiliations:** 1 Centers for Disease Control and Prevention, Kampala, Uganda; 2 Uganda Public Health Fellowship Program, National Institute of Public Health, Kampala, Uganda; 3 University of Oxford, Kellogg College, Oxford, UK; 4 Ministry of Health, Kampala, Uganda; 5 College of Health Sciences, Makerere University School of Public Health, Kampala, Uganda

**Keywords:** Field, Epidemiology, Training, COVID-19, Implementation

## Abstract

**Background:**

The global need for well-trained field epidemiologists has been underscored in the last decade in multiple pandemics, the most recent being COVID-19. Field Epidemiology Training Programs (FETPs) are in-service training programs that improve country capacities to respond to public health emergencies across different levels of the health system. Best practices for FETP implementation have been described previously. The Uganda Public Health Fellowship Program (PHFP), or Advanced-FETP in Uganda, is a two-year fellowship in field epidemiology funded by the U.S. Centers for Disease Control and situated in the Uganda National Institute of Public Health (UNIPH). We describe how specific attributes of the Uganda PHFP that are aligned with best practices enabled substantial contributions to the COVID-19 response in Uganda.

**Methods:**

We describe the PHFP in Uganda and review examples of how specific program characteristics facilitate integration with Ministry of Health needs and foster a strong response, using COVID-19 pandemic response activities as examples. We describe PHFP activities and outputs before and during the COVID-19 response and offer expert opinions about the impact of the program set-up on these outputs.

**Results:**

Unlike nearly all other Advanced FETPs in Africa, PHFP is delinked from an academic degree-granting program and enrolls only post-Master’s-degree fellows. This enables full-time, uninterrupted commitment of academically-trained fellows to public health response. Uganda’s PHFP has strong partner support in country, sufficient technical support from program staff, Ministry of Health (MoH), CDC, and partners, and full-time dedicated directorship from a well-respected MoH staff member. The PHFP is physically co-located inside the UNIPH with the emergency operations center (EOC), which provides a direct path for health alerts to be investigated by fellows. It has recognized value within the MoH, which integrates graduates into key MoH and partner positions. During February 2020-September 2021, PHFP fellows and graduates completed 67 major COVID-related projects. PHFP activities during the COVID-19 response were specifically requested by the MoH or by partners, or generated *de novo* by the program, and were supervised by all partners.

**Conclusion:**

Specific attributes of the PHFP enable effective service to the Ministry of Health in Uganda. Among the most important is the enrollment of post-graduate fellows, which leads to a high level of utilization of the program fellows by the Ministry of Health to fulfill real-time needs. Strong leadership and sufficient technical support permitted meaningful program outputs during COVID-19 pandemic response. Ensuring the inclusion of similar characteristics when implementing FETPs elsewhere may allow them to achieve a high level of impact.

## Background

Effective response to COVID-19 has challenged even the most well-resourced countries [1]. All affected countries struggled with coordination and with limited epidemiologic, clinical, and management capacities in surveillance, quarantine and isolation, infection prevention and control, case management, and data collection and management [1, 2]. While national leadership has often been charged with responsibility for the success – or failure - of individual country responses [3–5], less has been said about the importance of having the right human resources to fully support each national response.

The Field Epidemiology Training Program (FETP) was designed in part to provide human resources to address such situations. FETPs preceded the Global Health Security Agenda (GHSA), but are directly aligned with its efforts in workforce development [6]. Present in > 65 countries worldwide [7], FETPs build capacity at all levels of the public health system with three different programs aimed at district-level (‘Frontline’) [8], regional-level (‘Intermediate’) [9], and national-level (‘Advanced’) public health staff. These in-service training programs usually represent government-to-government collaborations between the U.S. CDC and the host country government. In such programs, initial funding and technical support for FETPs is provided by the U.S. Centers for Disease Control and Prevention, but some countries subsequently take over the funding in part or in full through national budgets or other donors [6]. Globally, Advanced FETPs are intensive two-year programs that train public health professionals, called residents or fellows, in integral aspects of disease response and control. Advanced FETP fellows are placed in government public health host sites during their two years, often at the district level, and are mentored by host site supervisors and program staff. They provide key services to their host sites while working with national-level staff to bolster preparedness, address outbreaks, and conduct field epidemiologic studies [6].

‘Best practices’ for FETP implementation have been described previously [10, 11]. Some of these include full-time participation by fellows, a maximum of classroom time (< 25% of the full program time), ownership of the program by the Ministry of Health, sufficient funding, high-quality supervision and sufficient mentorship, and the presence of a well-respected, full-time, local program director. However, each FETP is different, and the relative strengths of each program depend on multiple variables.

## Methods

We briefly describe the history of Uganda Advanced FETP, called the Public Health Fellowship Program (PHFP), as well as its recruitment strategies, leadership, partner affiliations, organization within the Ministry of Health and National Institute of Public Health, staffing, funding, technical support, and achievements during the COVID-19 pandemic. Data on fellows’ scientific activities and products, including COVID-19-related and other projects and publications, were abstracted from a program database used by PHFP staff to track fellows’ activities. We use COVID-19 response activities as examples to highlight how program characteristics combined to facilitate a strong COVID-19 response from PHFP, aligned with Ministry of Health (MoH) needs and priorities and producing meaningful outputs.

## Results

### History and description of PHFP in Uganda

Initiated in 2015, PHFP is a two-year, post-Master’s-degree field epidemiology training program. Between one-third and one-half of the fellows also hold a medical or veterinary degree; others are pharmacists, nutritionists, economists, statisticians, nurses, wildlife specialists, or others. The program is based in the Uganda National Institute of Public Health (UNIPH), located within the Ministry of Health (MoH), and funded by the U.S. CDC. The director of UNIPH also serves as the program director for the PHFP. A strong partnership with the Makerere University School of Public Health (MakSPH) and the U.S. CDC enables technical support to the program and partnerships on individual projects [12, 13].

Candidates for the fellowship apply in response to a nationwide newspaper advertisement placed in June of each year. Qualifying applicants are selected through a competitive process requiring written personal statements, letters of recommendation, academic paperwork, and interviews with a panel of program staff. In 2022, approximately 250 applications from around the country were reviewed. Program enrollment is aligned with the calendar year and cohorts are named by the year of entry; for example, Cohort 2022 (‘C2022’) began in January 2022 and will finish in December 2023. In-class coursework is conducted during eight weeks of January and February and two weeks in September during the first year. Exact timing is tailored around existing field needs, and training is shifted if a field response is ongoing.

In contrast to some other Advanced FETPs [6], all PHFP fellows in Uganda are placed at national-level host sites across the MoH in the capital city of Kampal﻿a. Close mentorship, individual work and teamwork, and time spent in the field at the local and regional levels conducting on-the-ground activities and responses are key characteristics of the program. Fellows must complete multiple competency-based deliverables during their time in the program, including in outbreak investigation/public health emergency response, surveillance data analysis, surveillance system evaluation, applied epidemiologic studies, cost analyses of outbreaks, quality improvement science, burden of disease estimation, and leadership skills [12, 13]. Field projects are selected based on MoH and other stakeholder priorities at the time. Despite the global health security nature of the work, almost all the funding comes from the U.S. government, via the President’s Emergency Plan for AIDS Relief (PEPFAR) and from the President’s Malaria Initiative (PMI). As a result, all PHFP fellows carry out HIV- and TB-related priority projects related to PEPFAR targets as well as malaria-related activities [13]. As of September 2021, PHFP fellows had completed 191 outbreak investigations, 370 epidemiologic studies and quality improvement projects, and published 88 manuscripts in peer-reviewed journals and 189 public health bulletin articles from their work in the program. They have also given national and international presentations on their work, written policy briefs, conducted trainings and assessments, and led preparedness activities. Since inception, the program has graduated 65 field epidemiologists, many of whom now hold key positions in the Uganda public health system. An additional 29 fellows are in training as of January 2022. PHFP is recognized as one of the strongest FETPs in Africa and the program has garnered multiple awards. The program and many of its pre-COVID-19 outputs have been described in detail elsewhere [12].

### Activities of the PHFP fellows and graduates during the COVID-19 response

The emergence and global spread of COVID-19 in early 2020 coincided with the start of the classroom training for Cohort 2020. As a result, the didactic training for this cohort was easily tailored to COVID-19 and provided readings, examples, and discussions about the disease two months before it arrived in Uganda. Shortly after the first cases were identified in Uganda, PHFP staff discussed activities that could both fulfil the PHFP fellowship deliverables and meet the needs of a rapidly-evolving response. From February 2020 through September 2021, PHFP fellows and graduates carried out 67 major COVID-19 response activities (Table 1). These represented a combination of activities requested by the MoH and other stakeholders, and those generated *de novo* by the program. Many provided real-time data to inform ongoing response. This report will focus on specific characteristics of PHFP that made it particularly well-suited as an FETP to engage effectively in the COVID-19 response in Uganda.

**Table 1:** Activities carried out by Public Health Fellowship Program fellows and graduates to support the COVID-19 response in Uganda, 2020–2021. PHFP cohorts are listed by the year of entry under ‘project investigators’

#	Date	Activity	Project investigators	Outputs as of November 2021
1	February 2020	Setting up, conducting traveler screening at Entebbe International Airport^a^	C2019, C2020	1, 3, 4 (in preparation), 6
2	March-June 2020	Use of a toll-free call center for COVID-19 response and continuity of essential services during the lockdown in Greater Kampala, Uganda, 2020	C2020	4 (under review [14])
3	March – September 2020	Setting up and overseeing contact tracing team; management of contact tracing data; analysis and daily presentation of surveillance data^a^	C2018	1, 2
4	March-Oct 2020	Contact tracing nationwide	C2018, C2019, C2020	2
5	March-December 2020	Evaluating spatiotemporal incidence of COVID-19 in Uganda	C2018	2,
6	March 2020-present	Analysis and management of clinical data for case management of COVID-19 patients	C2020	1, 2
7	April 2020	Access to food and essential medicines among Ugandans during the COVID-19 lockdown: a cross-sectional study (online survey)	C2020	3, 4 [15]
8	April 2020	Survey of violence and discrimination among Ugandan residents during the COVID-19 lockdown in Uganda	C2020	4 [16]
9	April 2020	Health facility operational readiness assessment for COVID-19 in Kampala and Wakiso Districts	C2019, C2020	1, 2, 3
10	April 2020	Risk mapping, population movement, and connectivity across border districts in southern Uganda during the COVID-19 pandemic	C2019/C2020	2, 3, 4 (in preparation)
11	April-May 2020	Analysis of transmission from early cases of COVID-19 in Uganda	C2020, C2018	1, 3, 4 (Migisha, 2020 #2)
12	April-May 2020	Burden and psychological impact of COVID-19 among healthcare workers in Uganda	C2020	3, 4 [17]
13	April-May 2020	Survey to assess the association between perceived risk of COVID-19 and protective behavior	C2020	3, 4 (in preparation)
14	April-May 2020	Level and determinants of adherence to COVID-19 prevention measures during the first stage of the outbreak	C2020	3, 4 [18]
15	May 2020	Setup and management support for institutional quarantine centers in Masindi District	C2020	2
16	May 2020	Regional risk mapping and building capacity for COVID-19 preparedness and response in Mbale region (16 districts).	C2018	2
17	May 2020	Quantitative and qualitative evaluation of factors associated with COVID-19 infection among truckers testing positive at the Ugandan border	C2020	1, 2, 3, 4 [19]
18	May 2020	Investigation of COVID-19 outbreak in Masindi District	C2020	1, 2
19	May 2020	Investigating family clusters of COVID-19 in Eastern Uganda	C2018	1, 2
20	May, 2020	Contact Tracing and Community-Based Surveillance for COVID-19 Using Health Assistants, Masindi District, Uganda	C2020	3, 4 (under review)
21	May 2020	Using air quality levels to measure adherence to COVID-19 lockdown in Kampala	C2020	2, 3, 4 (in preparation)
22	May – June 2020	National KAP survey about COVID-19 in Uganda	C2020	4 (in preparation)
23	May 2020 – May 2021	Cost-effectiveness evaluation of national airport screening policy options for COVID-19 in Uganda	C2020	3, 4 [20]
24	May 2020-present	Surveillance and analysis of data for healthcare worker COVID-19 infections in Uganda	C2017	1, 2, 4 (in preparation)
25	May-June 2020	Community assessment of COVID-19 transmission in fishing communities in Kasensero, a high-risk boat landing site on Lake Victoria	C2019	2, 3, 4 (in preparation)
26	July 2020	KAP study for COVID-19 in Nakivale Refugee Settlement	C2020	3, 4 (under review)
27	July 2020	Using a human-centered design approach to increase uptake of COVID-19 prevention measures in informal settlements in Kampala	C2020	3, 4 (in preparation)
28	July 2020	Evaluating compliance to COVID-19 prevention measures in Kampala to inform phased lifting of the lockdown	C2020	2, 3
29	July-August 2020	Evaluation of risk factors for gender-based violence during the COVID-19 lockdown in Kampala, Lira, and Gulu Districts	C2020	4 (in preparation), 5 [21]
30	August 2020	Investigation of community COVID-19 cases in South-Western Uganda	C2020	1, 2
31	August 2020	Investigation of COVID-19 cases in Fort Portal and Mubende isolation centers	C2020	1
32	August 2020	Epidemiological investigation of COVID-19 cases and response to COVID-19 in Mbale Region	C2020	2
33	August 2020-present	Comorbidities and risk factors for poor outcomes among hospitalized patients with COVID-19 in Uganda	C2020	1, 2, 4 (in preparation)
34	August-September 2020	Investigation of community COVID-19 cases in Gulu and Lira	C2018, C2019, C2020	1
35	August 2020	Outbreak investigation of COVID-19 in Amuru Prison	C2019, C2020	1
36	August 2020	Outbreak investigation of COVID-19 at Gulu Prison	C2019, C2020	1
37	July 2020	Investigation of low district reporting rates for COVID-19 and implementing improvement approaches	C2020	1, 3
38	August 2020	Impact of COVID-19 on health service delivery in Uganda	C2020	3, 4 (in preparation)
39	September 2020	Evaluation of factors associated with mental and psychosocial wellbeing of healthcare workers in refugee settings during the pandemic	C2020	4 (in preparation)
40	September, 2020	Investigation of COVID-19 cases in West Nile region	C2018	2
41	September 2020	Outbreak investigation of COVID-19 cases in Abim District	C2020	2, 4 (in preparation)
42	September 2020	Impact of COVID-19 on care and management of diabetes in Uganda	C2020	4 (in preparation)
43	October 2020	Investigation of COVID-19 outbreak at hydropower plant	C2020	2, 4 (under review)
44	October 2020	Epidemiological assessment of COVID-19 cluster among attendees of a church activity in Omoro District, Northern Uganda	C2020	4 (under review), 5 [22]
45	October 2020	Outbreak investigation of COVID-19 among factory workers at Factory X in Buikwe district	C2020	2, 4 (in preparation)
46	October 2020	COVID-19 outbreak investigation in Moroto Prisons	C2020	2, 4 [23]
47	October 2020	Investigation of COVID-19 outbreak in Masaka Saaza Prison	C2019, C2020	2, 4 (in preparation)
48	October – November 2020	COVID-19 in East Africa and Democratic Republic of Congo: A Comparison of the Outbreaks and Interventions in the First Four Months	C2019	
49	November 2020	Rapid Assessment of COVID-19 response in Kiryandongo district following upsurge in cases and deaths	C2018	1, 2
50	November 2020	Estimating the cost of managing COVID-19 patients in Uganda early during the outbreak	C2020	4 (in preparation)
51	November 2020	Investigation of outbreak and factors associated with SARS-CoV-2 infection among students, teachers and support staff at a secondary school in Kampala, Uganda	C2020	1, 2, 4 (in preparation)
52	December 2020	Epidemiologic investigation of household and individual risk factors for infection among household members of COVID-19 patients in home-based care in Western Uganda	C2020	2, 4 (under review) [24]
53	December 2020	Changes in human movement patterns along the Uganda-DRC border in response to the COVID-19 lockdown	C2018	2
54	January 2021	Evaluating association between political campaign activities and COVID-19 upsurges	C2020	2
55	February 2021	Leveraging COVID-19 resources for EVD preparedness in Kasese District	C2018	2, 4 (in preparation)
56	February 2021	Knowledge, perceptions and barriers around uptake of COVID-19 vaccine in Uganda, 2021	C2020	2, 4 (under review)
57	March-April 2021	Investigation of preventable factors associated with COVID-19 patient deaths among persons hospitalized in Greater Kampala	C2019, C2020, C2021	1, 4 [25]
58	April 2021	Investigation and response to a cluster of COVID-19 cases in Gulu, Kitgum and Kiryandongo Districts	C2017, C2018	1,2
59	April-June 2021	Evaluation of impact of COVID-19 test burden on turnaround time for HIV viral load and early infant diagnosis testing	C2020	1, 2, 4 (in preparation)
60	April 2021 - present	Providing technical support to districts on Uganda COVID-19 vaccination eRegistry (DHIS2 system)	C2021	5
61	May 2021	Description of adverse events following immunization in Uganda	C2021	2, 4 (in preparation)
62	May-June 2021	Evaluation of readiness and preparedness of health facilities in Uganda to manage the second wave of COVID-19	C2021	1, 2, 4 [26]
63	June 2021	Comparing clinical characteristics and vaccination status of hospitalized and non-hospitalized cases of COVID-19 during the second wave of COVID-19 with previous waves to inform messaging for risk groups and vaccination	C2021	1, 4 [27]
64	July 2021	Post-acute COVID-19 syndrome among persons hospitalized at two national referral hospitals	C2021	1, 2, 4 (in preparation)
65	August 2021	Evaluation of performance of laboratory services during the COVID-19 response in Uganda	C2021	1, 2
66	September 2021	Investigation of an outbreak of COVID-19 among patients in a psychiatric unit	C2020	2, 4 (in preparation)
67	September 2021	Investigation of an outbreak of COVID-19 in pediatric and neonatal hospital wards	C2020	2, 4 (in preparation)

1 Presentation(s) to Incident Management Team

2 Presentation(s) to other relevant teams (District Health teams Uganda Prisons Service, COVID-19 response pillar teams, EOC, etc.)

3 International / domestic conference presentations

4 Manuscripts in preparation, submitted, in press, or published (if published, citation included)

5 Other, including UNIPH epidemiologic bulletin articles (citations included)

^a^Collaboration between PHFP fellows or graduates and other staff at MoH or partners

### Post-Master’s degree program

In most Advanced FETPs, fellows complete a Master’s Degree in Public Health (MPH) with a program-affiliated university over the two years while also conducting their field work [6, 7, 10]. In contrast to all but one other FETP in Africa (the Sudan FETP) (J. Harris, personal communication), the Uganda PHFP accepts only post-Master’s-degree candidates and does not award a degree. As a result, PHFP represents something more akin to a medical residency than an academic program. Because it is unlinked to a degree-granting program, fellows have almost the full two years of the program to spend on fieldwork, undisrupted by academic coursework or scheduling. When training is required, scheduling is flexible.

During COVID-19, this had two benefits: first, having already completed their Master’s Degrees in Public Health (or a related field), fellows already knew the didactic aspects of outbreak investigation and control, and no valuable time was lost bringing them ‘up to speed.’ Supervisors were able to immediately deploy and mentor fellows, rather than teach them new academic information, during these activities. Second, fellows could prioritize their field activities completely without concern about classes, exams, or thesis work. These factors have increased the value of the PHFP for the MoH both before and during the COVID-19 response.

### Strong level of MoH recognition/utilization

While there is no specific post-PHFP job track for graduates in Uganda, the program is well-recognized by the MoH. Fellows are utilized by the MoH for many activities, and most graduates are retained by the MoH and partners after graduation. Starting in mid-2021, epidemiologist positions were opened at seven of the 14 regional referral hospitals nationwide, and some have been filled by some of the program graduates (J. Harris, personal communication). As of September 2022, 46 of the 64 graduates with known occupations work for the Ministry of Health or its partners in a variety of positions (Table 2).

**Table 2:** Positions of PHFP graduates with known positions as of September 2022 (of 65 graduates)

Employer	# of graduates	Position(s)
MoH and partners^a^	46	Epidemiologists, biostatistician, district health and veterinary officers, OneHealth coordinator, program officers, medical officer, laboratorian; PHFP staff; Frontline FETP Resident Advisor, Intermediate FETP Resident Advisor & staff
Academia	3	
Africa CDC outside Uganda	3	epidemiologist
CDC Uganda	3	Outbreak coordinator, COVID vaccine program officer, COVID-19 funding program officer
Other	4	Epidemiologist, biostatistician
WHO outside Uganda	2	epidemiologists
CHAI	1	epidemiologist
**TOTAL**	**64**	

^a^Includes District Health Offices, Regional Emergency Operations Center, Ministry of Agriculture, Animal Industry, and Fisheries, Mildmay, Baylor Uganda, Infectious Diseases Institute, WHO Uganda, Kampala Capital City Authority, AFENET, International Rescue Committee, Task Force for Global Health

PHFP fellows and graduates in Uganda are seen as a strong human resource during public health emergencies. During the COVID-19 pandemic, many graduates were placed in key response roles by the MoH. Because fellows are hosted at MoH sites during their fellowship, specific skill sets of different graduates were well-known to the MoH staff and were utilized during the COVID-19 response. Projects overseen by graduates included rollout and management of contact tracing and data management, healthcare worker infection surveillance, risk mapping, supervision of teams deployed to investigate outbreaks, and others. The COVID-19 Incident Management Team (IMT) conducted regular outreach to current fellows to support projects of interest, including investigation of preventable causes of COVID-19 deaths, investigation of clusters of COVID-19 in congregate settings such as prisons [23], hospitals, and schools, health facility preparedness assessments [26, 28], and investigations of COVID-19 outbreaks in home-based care settings [24] (Table 1).

### Sufficient technical support within and outside of the PHFP program staff

Having sufficient technical support and mentors for the number of fellows available is a key component to the success of any FETP. Starting in 2017, PHFP hired three graduates from its first cohort of 10 fellows who form three of the five key technical staff persons supporting PHFP fellows. All three have been retained as full-time staff, with one scientific writer, one field supervisor, and a training manager [29]. At the beginning of 2022, the program director took on a greater leadership role with the UNIPH, and one of the two field supervisors was promoted to be program coordinator. At that time, a graduate of Cohort 2020 was hired to serve as the second field supervisor. The program also has a resident advisor from CDC who lives in Uganda and provides hands-on technical support to the program [29]. Despite their different job titles, PHFP staff often work interchangeably across roles. The longevity of the staff members with the program provides benefits in terms of meeting program needs, including recognition with key public health personnel, an in-depth understanding of the public health system throughout Uganda, knowledge about how to access datasets and conduct administrative activities, and a high level of technical competency. Beyond this, the program receives technical support from many CDC staff, other stakeholder staff, and in-country graduates, who provide a large pool of persons to request, supervise, and facilitate epidemiologic work.

During the COVID-19 response, having this breadth of support outside of the regular program staff was instrumental in implementing quality projects. One example included a project to reduce COVID-19 risk for cargo truck drivers during the COVID-19 outbreak, who served as the primary source of COVID-19 infections for the first few months of the epidemic in Uganda [30]. PHFP embarked on a project to identify specific locations at the border and inside the country where truckers were likely to have high-risk exposures to COVID-19. The project was partly supervised by CDC and MoH staff with a history of identifying approaches to reduce HIV in truck drivers; their access to leadership within cargo truck driving organizations and knowledge of cargo truck driver culture were key in carrying out a successful project and informing appropriate interventions.

### Dedicated directorship

All FETPs have a program director, usually located at the MoH. However, having a program director in place does not by itself guarantee program success. Programs require advocacy for inclusion of fellows in multiple activities as well as placement within the MoH [6]. The Uganda PHFP has a physician-scientist program director with a high level of recognition and respect within the MoH. The program director also heads the UNIPH, for which the mandate is directly aligned with fellows’ activities. The director’s extensive history of scientific and programmatic collaboration with the MoH and partners also facilitates awareness of the program within the MoH. During the COVID-19 pandemic, the program director advocated for the fellows’ involvement in many aspects of the response. This enabled a much broader array of work for the fellows beyond the more generalized (but still critical) response activities of contact tracing and surveillance.

### Program placement with the EOC

The UNIPH currently houses PHFP, FETP-Frontline, FETP-Intermediate, and the Public Health Emergency Operations Center (EOC) [12, 31–33]. The EOC in Uganda is responsible for coordinating information and resources (human and physical), as well as organizing, conducting, and managing all aspects of public health emergency response [33]. Within the UNIPH, EOC and PHFP (as well as the other levels of FETP) are located on the same floor and there is substantial communication between the programs. This co-location is key in facilitating effective sharing of data about outbreaks, including COVID-19, and collaboration on rapid response teams. During the start of the COVID-19 outbreak, the national COVID-19 call center was activated through the EOC. Surveillance data were tracked by the EOC, and the continued tracking of other diseases and outbreaks took place at the EOC. All COVID-19 IMT meetings were hosted at the EOC, as are national task force meetings about other outbreaks, and thus there is no physical barrier to the participation of fellows in such meetings. As countries ramp up their own efforts to build National Institutes of Public Health (also called National Public Health Institutes), co-locating these two arms of public health response can facilitate effective public health emergency response.

### Strong implementing partners and partnerships

In Uganda, regional implementing partners (IPs) associated with PEPFAR cover non-overlapping areas of the country to carry out HIV-related activities [34]. For several years, PHFP has been working directly with IPs to achieve their HIV project deliverables. As a result, PHFP is well-known to these partners. When hotspots of COVID-19 arose in different areas, some IPs requested PHFP fellows to support their response. In one example, an IP for the MoH raised an alert about a large upsurge of COVID-19 cases among household members of persons in home-based care in their catchment area. The connection between the IP, PHFP, the MoH team responsible for home-based care facilitated rapid action and response [24]. The investigation identified key individual and led to revised home-based care guidelines to reduce risk of COVID-19 among household members of persons in home-based care [35].

Beyond the PEPFAR IPs, the program has a strong partnership with the MakSPH, which in turn has strong partnerships with many external partners. During the COVID-19 outbreak, MakSPH supported the program to carry out many projects. One example included supporting fellows to conduct national surveys, using tools developed by a collaboration of international scientists, on experiences of multiple countries during the pandemic. Fellows modified the protocol and survey tools to suit the Ugandan context and received institutional review board approval to assess both data on violence and discrimination during the COVID-19 lockdown in Uganda and determinants of adherence to preventive measures early in the COVID-19 outbreak [16, 18]. The school also provided the idea and technical supervision for a study using a human-centered design approach to increase uptake of COVID-19 prevention measures in informal settlements in Kampala (manuscript in preparation). The expertise provided by the school led to a high-quality project outside the normal scope of expertise among the PHFP staff.

### Experience

Uganda experiences dozens of outbreaks each year, ranging from relatively more benign conditions to viral hemorrhagic fevers. As a result, both fellows and staff have extensive experience in outbreak investigations and the associated administrative and political impact. Key outbreak preparedness and response activities have included those for Ebola virus disease [36, 37], Marburg virus [37], plague, anthrax [38–40], measles [41–46], yellow fever [47], Rift Valley fever, malaria [48–50], typhoid, Crimean-Congo hemorrhagic fever [51], food poisoning [52, 53], chemical poisoning [54, 55], rabies [56], typhoid [57], leprosy [58], cholera [59–61], tuberculosis [62], podoconiosis [63], and many others [12]. These investigations have occurred countrywide and, at times, in collaboration with neighboring countries [52]. Early issues with rapid response and administrative challenges have been smoothed out over the years. During the COVID-19 outbreak, this level of experience has facilitated the ability to move quickly and effectively in the field, with fewer logistical and administrative snags than might have otherwise been encountered.

## COVID-related projects carried out the PHFP during the pandemic

Because PHFP represents the bulk of the MoH’s trained field epidemiology workforce [64], it is viewed by the MoH as having primary responsibility for field epidemiology investigations in Uganda. While many of the programmatic activities listed in Table 1, such as airport screening, contact tracing, and setup of quarantine and call centers would almost certainly have occurred without PHFP support, the outbreak investigations, risk assessments, KAP studies, population movement evaluations, transmission studies, studies about the of vaccine and other epidemiologic studies are unlikely to have been done without PHFP. In general, these studies were directly requested by the MoH or partners to provide critical data to rapidly inform the response. Additional examples beyond those above include the multiple outbreak investigations in prisons, which provided real-time evidence of the primacy of mask use over other interventions in prison settings in protecting prisoners against COVID-19 [23]. These data were used to make recommendations to prisons to use resources to ensure all prisoners had access to and used masks. A nationwide health facility assessment in mid-2021 demonstrated major gaps in preparedness as well as failures to adhere to infection prevention and control, lack of space for COVID-19 patients, insufficient lifesaving supplies, and lack of case reporting [26, 28]. The findings were presented in real-time to the COVID-19 IMT at the Ministry of Health, allowing them to immediately identify additional space and staff for overstretched facilities and implement new reporting approaches to enable accurate case counts (P. Mwine, unpublished data). Case management and surveillance data, both of which were partially supported by PHFP fellows and graduates, were presented daily at the IMT meetings for action. Studies were used to address public rumors, as well. Pursuant to social media rumors that fully vaccinated persons were dying of COVID-19 in hospitals in Uganda (shortly after vaccine rollout), PHFP conducted a study comparing the epidemiology of COVID-19 cases between different waves of infection and included vaccination as a cofactor. Among other findings from the study of 800 hospitalized and non-hospitalized persons across the first and second COVID-19 waves in Uganda, the authors demonstrated that none of the 400 hospitalized patients in the study had been fully vaccinated, and 94% had not received any doses of vaccine [27]. The MoH publicized the study results through social media platforms to reassure the public and promote the need for vaccination. The capacity to carry out such activities and inform responses in real time are crucial to a strong pandemic response.

## Discussion

The PHFP (Advanced FETP) in Uganda was able to provide robust and complementary support to the national COVID-19 response. This support was facilitated by specific noteworthy characteristics of the Uganda PHFP, including being a non-degree-granting program, having a high level of recognition within the MoH, having sufficient technical and administrative support within and outside of the MoH, being placed together with the EOC, having a well-recognized and well-respected director, and an extensive history with many different field investigations. While many of these are not unique to PHFP, the combination of all of these factors together facilitated a stronger response and utilization of the program as it was meant to be used.

Having a non-degree-granting program may be the single most important factor in facilitating the quality and quantity of outbreak response activities during COVID-19. Entry into the program already armed with basic knowledge of epidemiology (including outbreak investigation) and biostatistics, combined with the lack of competition with academic coursework, enables complete dedication to fieldwork and a basic level of academic competency. However, this is very rare among FETPs, primarily because non-degree-granting training programs – especially long-term programs such as Advanced FETPs - can face challenges with social acceptance. The primary barrier involves the reluctance of potential enrollees to spend two years in a program that does not award a degree, as it might not advance them in the traditional employment system. Two factors are key in addressing this issue: first, recognition and valuing of the program by the MoH is essential. Fellows need to know that their experience in and graduation from the program will enable them to obtain a better position than they otherwise could with their Master’s degree. It is particularly helpful for countries with FETPs to have a dedicated post-program job track for graduates. While a PHFP-specific job track does not exist in Uganda, epidemiologists are valued throughout the MoH and with other stakeholders. As a result, fellows are typically quickly placed in jobs after graduation. Thus, this represents a self-reinforcing cycle: ministries of health may value the program more when fellows have already completed a degree – and tend to see them less as ‘students’ and more as ‘junior staff’ or ‘residents’ - and fellows are better positioned to respond effectively when they already have the academic training required to execute many public health activities.

Second, compensating the fellows in a manner that is fair for their level of education while recognizing the fact that they are in an in-service training program is critical for a non-degree-granting program. Inadequate compensation will fail to recruit strong candidates who could otherwise be employed with their degree, while excessive compensation will draw applicants for the wrong reasons. In Uganda, fellows receive a small stipend plus benefits, representing a salary that is on the lower end for post-Master’s-degree candidates but highly ‘livable’ in Uganda. Similarly, officers in the Epidemic Intelligence Service (EIS) at CDC, after which FETPs are modeled, earn salaries that are on the lower end for their educational level but provide adequately for a good quality of life during the program [65]. Money that is saved in paying tuition for fellows to achieve an MPH can thus be used to compensate post-degree fellows fairly.

Beyond having a non-degree-granting program, having sufficient technical support for FETP fellows is important to their success both during pandemics and during ‘normal’ times. In EIS, the ratio of supervisors to EIS officers is extremely high: each officer is supervised by or has access to supervision by many doctoral-level staff [66]. In most FETPs, the opposite is true: the number of trainees far exceeds the number of technical staff that provide support. Few Advanced-level FETPs have the level of support available to the Ugandan PHFP fellows, from experts at the MoH, CDC, MakSPH, and implementing partners. This is in part due to the long-term engagement of staff in the program; however, it also reflects the strong level of program recognition and engagement by all partners, including non-governmental implementing partners, and inclusion of the fellows in their programming. Again, this is partially reflected in the level of experience with which PHFP fellows enter the program and their perceived value more as an existing workforce than as students.

PHFP activities in the response did not always run smoothly. Unlike many programs, PHFP fellows are all placed at the national level. This facilitates a high level of national engagement but can present barriers to provision of ongoing support to regional or district levels. Ideally, FETP-Frontline and FETP-Intermediate are meant to address this issue, but neither were active during the first year of the pandemic (though both are actively training in 2021). In addition, at times there were challenges with availability of fellows or their ability to present their field reports in a timely fashion, often due to their work on multiple response projects simultaneously. Work on other, non-COVID-related projects that normally form part of the PHFP portfolio, including HIV projects and investigations of endemic diseases, was challenged. Despite these challenges, from the inception of the epidemic through September 2021, the PHFP provided extensive service to the COVID-19 response in Uganda. The program will continue to learn from the COVID-19 epidemic response experience and seek to continually improve both during pandemic and non-pandemic times.

Because few Advanced-level FETPs publish on their program organization or summarize their outputs, we cannot directly compare the Uganda PHFP to other Advanced-level FETPs to prove that the characteristics of PHFP make a difference in the program’s success. Indeed, the most recent multisite evaluation of FETP-Advanced programs was in 2014 and included only 10 sites, most outside of Africa [10]. While we acknowledge the limitations of our approach in evaluating the characteristics of PHFP, it is our hope that this paper can display how some of the best practices in FETP program set-up and management can translate into real-world success for a field epidemiology training program. 

## Conclusion

The program model used by the Uganda PHFP enables it to effectively address emerging and re-emerging health threats and global health security needs. Enrollment of post-Master’s-degree fellows, strong support from MoH leadership and partners, strong program directorship, and integration of PHFP within MoH and with EOC are aligned with best practices for FETPs and enable a productive program aligned with interests of the Ministry of Health. Consideration of these characteristics when implementing FETPs elsewhere could help facilitate program effectiveness, both during non-pandemic periods and during epidemic response.

## Data Availability

All data generated or analyzed during this study are included in this published article.
